# Hadrontherapy Interactions in Molecular and Cellular Biology

**DOI:** 10.3390/ijms21010133

**Published:** 2019-12-24

**Authors:** Juliette Thariat, Samuel Valable, Carine Laurent, Siamak Haghdoost, Elodie A. Pérès, Myriam Bernaudin, François Sichel, Paul Lesueur, Mathieu Césaire, Edwige Petit, Aurélie E. Ferré, Yannick Saintigny, Sven Skog, Mihaela Tudor, Michael Gérard, Sebastien Thureau, Jean-Louis Habrand, Jacques Balosso, François Chevalier

**Affiliations:** 1 Department of Radiation Oncology, Centre François Baclesse, 14000 Caen, France; jthariat@gmail.com (J.T.); paul.lesueur89@gmail.com (P.L.); mathieu.cesaire@hotmail.fr (M.C.); m.gerard@baclesse.unicancer.fr (M.G.); jl.habrand@baclesse.unicancer.fr (J.-L.H.); j.balosso@baclesse.unicancer.fr (J.B.); 2 Laboratoire de Physique Corpusculaire IN2P3/ENSICAEN-UMR6534-Unicaen-Normandie Université, 14000 Caen, France; sebastien.thureau@chb.unicancer.fr; 3 ARCHADE Research Community, 14000 Caen, France; samuel.valable@cnrs.fr (S.V.); carine.laurent@unicaen.fr (C.L.); siamak.haghdoost@ganil.fr (S.H.); peres@cyceron.fr (E.A.P.); bernaudin@cyceron.Fr (M.B.); francois.sichel@unicaen.fr (F.S.); epetit@cyceron.Fr (E.P.); ferre@cyceron.fr (A.E.F.); yannick.santigny@cea.fr (Y.S.); 4 Normandie Univ, UNICAEN, CEA, CNRS, ISTCT/CERVOxy Group, GIP CYCERON, 14000 Caen, France; 5 Normandie Univ, UNICAEN, UNIROUEN, ABTE, 14000 Caen, France; 6 LARIA, iRCM, François Jacob Institute, DRF-CEA, 14000 Caen, France; 7 UMR6252 CIMAP, CEA-CNRS-ENSICAEN-Université de Caen Normandie, 14000 Caen, France; mihaela.tudor@nipne.ro; 8 Sino-Swed Molecular Bio-Medicine Research Institute, Shenzhen 518057, China; svenisak@icloud.com; 9 Department of Life and Environmental Physics, Horia Hulubei National Institute of Physics and Nuclear Engineering, PO Box MG-63, 077125 Magurele, Romania; 10 Faculty of Biology, University of Bucharest, Splaiul Independentei 91-95, R-050095 Bucharest, Romania; 11 Department of Radiation Oncology, Centre Henri Becquerel, 76000 Rouen, France

**Keywords:** cancer, radioresistance, particle therapy, hadrontherapy, hypoxia

## Abstract

The resistance of cancer cells to radiotherapy is a major issue in the curative treatment of cancer patients. This resistance can be intrinsic or acquired after irradiation and has various definitions, depending on the endpoint that is chosen in assessing the response to radiation. This phenomenon might be strengthened by the radiosensitivity of surrounding healthy tissues. Sensitive organs near the tumor that is to be treated can be affected by direct irradiation or experience nontargeted reactions, leading to early or late effects that disrupt the quality of life of patients. For several decades, new modalities of irradiation that involve accelerated particles have been available, such as proton therapy and carbon therapy, raising the possibility of specifically targeting the tumor volume. The goal of this review is to examine the up-to-date radiobiological and clinical aspects of hadrontherapy, a discipline that is maturing, with promising applications. We first describe the physical and biological advantages of particles and their application in cancer treatment. The contribution of the microenvironment and surrounding healthy tissues to tumor radioresistance is then discussed, in relation to imaging and accurate visualization of potentially resistant hypoxic areas using dedicated markers, to identify patients and tumors that could benefit from hadrontherapy over conventional irradiation. Finally, we consider combined treatment strategies to improve the particle therapy of radioresistant cancers.

## 1. Introduction: Radioresistance and Radiocurability in Radiotherapy

Radioresistance has no consensus definition and varies between radiobiologists and clinicians. The radiobiological response of cells to irradiation has historically been defined using clonogenic survival curves, which represent the survival of a cell line as a function of the absorbed dose in vitro or in vivo [1,2]. Radiation-induced cell death, depending on the radiation dose, is usually quantified using the linear quadratic (LQ) model, which describes radiation-induced cell death as a combination of single-hit (lethal) and multi-hit (sublethal) events [3]. Radioresistant cell lines are defined by the surviving fraction of cells at 2 Gy clearly above 50%, but this parameter alone, determined at a relatively low dose, fails to fully characterize cellular radioresistance. 

Moreover, this mathematical representation, derived from cellular data, does not take into account tissue complexity. Further characterization of radioresistance includes—but is not limited to—kinetics, quantity, and type of unrepaired lesions, description of cell death types other than mitotic cell death, and the effects of the microenvironment and tumor genomics. Radioresistance has also been evaluated at the tissue level, using the TCD50 (tumor control dose 50%), the dose that permanently eradicates 50% of tumors of a defined type. 

Ultimately, clinicians use empirically defined doses and fractionation schemes (as initially defined by Claudius Regaud in 1922) to treat tumors while maintaining acceptable toxicity to normal tissue. Thus, a practical definition of radioresistance is closely related to the dose that is needed in routine practice to achieve radiocurability. Although dose is not the sole factor in the tumor response or relapse, tumors that require doses of 60 Gy or more are usually considered radioresistant. Clinically, radioresistance is assessed through the radiation-induced tumor response within weeks or months of radiotherapy or through radiocurability—i.e., the probability that a tumor will be cured with radiotherapy (which theoretically requires a follow-up of greater than 5 years), as assessed by tumor control probability (TCP). One additional level of complexity is that radioresistance depends on the capacity to deliver such doses, which is limited by the tolerance of radiosensitive tissues nearby and the capacity of the radiation therapy technique to provide adequate physical dose coverage to the tumor with sufficient radiobiological efficacy. 

**Figure 1:**
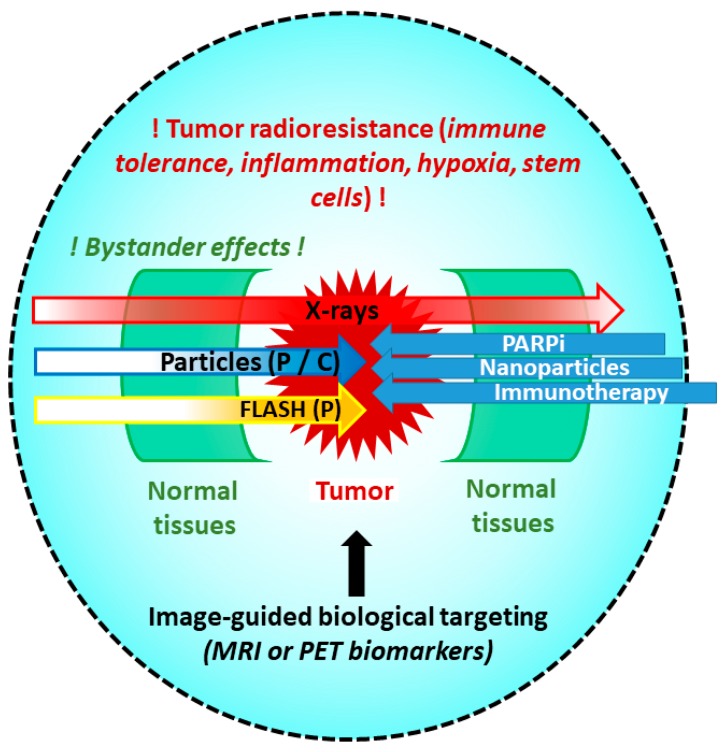
The radioresistance of cancer cells is a multifaceted mechanism, depending on the tumor type, location, and microenvironment. Radiosensitive organs near the tumor limit the irradiation dose using x-rays, but the use of particles (proton or carbon) can protect these normal tissues. In addition, carbon ions and FLASH irradiation improve the biological effect on the tumor, and combinations (PARPi, nanoparticle, immunotherapy) expand the possibilities of treatment. Image-guided radiotherapy increases the accuracy of the irradiation area and doses using biomarkers of radioresistant regions (hypoxia or stem cell niche).

For many tumors, current radiotherapy (RT) using photons or protons yields insufficient benefits with regard to local control and survival. One hypothesis is that these malignancies are radioresistant to low linear energy transfer (LET in keV/µm) radiation due to intrinsic characteristics, such as hypoxia. Carbon ion RT, a high-LET hadrontherapy, has had favorable results for radioresistant tumors compared with conventional RT. Various forms of hadrontherapy, possibly as combinations of ions, can be used to preserve healthy tissue and increase the antitumor efficacy in radioresistant tumors. Although hadrontherapy can spare normal tissues, based on their physical characteristics, the original modes of delivering radiation therapy with different particles—charged or neutral (photons)—have been investigated for approximately 25 years and are maturing in the clinical and preclinical phases and in animal models. Several of these advances are discussed below (Figure 1).

## 2. Optimized Forms of Radiation Therapy to Overcome Radioresistance

### 2.1. Proton Therapy in Tumor Control

In contrast to conventional radiotherapy using photons, proton therapy (PT) uses charged particles that are roughly 2000 times heavier than electrons. PT can be used to: (i) reduce the exposure of healthy normal tissues, and thus, lower the risk of toxicity, without any attempt to increase the dose to the target, and (ii) increase the dose to the target volume to improve the likelihood of tumor control, while maintaining doses under the limits of tolerance in surrounding tissues. 

These advantages are related primarily to the physical interactions of accelerated particles with matter: unlike x-rays (photons), which attenuate exponentially, with inevitable dose-deposition along their path, particles (such as protons and carbon ions) have a finite range and deposit most of their energy at the end of their range, commonly described as the “Bragg peak” (BP). These properties translate into a sharp decline in doses beyond the target, and no “exit” doses. 

PT has been used since the late 1950s for rare malignancies that were radioresistant, such as ocular melanomas and skull-base sarcomas. Such cancer locations were in close proximity to critical anatomical structures, which prevented radical resection, which is otherwise highly mutilating, and proper dose coverage using conventional (i.e., x-ray) irradiation. PT has become the standard practice for ocular melanomas since the 1990s, effecting > 95% local control [4] and ≥ 75% eye preservation rates [5]. Conventional x-ray irradiation of skull base tumors has been limited to infracurative doses of 60 Gy due to the proximity of critical structures (primarily the optic pathway and brain stem). Safely delivered proton doses of 70 Gy CGE [cobalt Gray equivalent = physical dose × estimated mean 1.1 RBE (relative biological efficiency), now specified as “Gy (RBE)”] reproducibly achieved >70% 5-year local control using passively delivered PT compared with ≤ 40% with conventional irradiation [6,7]. Outcomes with radioresistant and poorly limited, rapidly proliferating glioblastomas have been less favorable. 

Data on tumor progression in areas that have received less than 70 Gy (RBE) [8] have not been reproduced; however, higher doses are highly toxic to the brain [9]. The introduction of rotating gantries in PT in the mid-1990s and, more recently, the active scanning mode has made common extracranial tumors more accessible to PT. 

The spatial distribution of protons thus allows efficient irradiation of radioresistant tumors, but mature results and randomized trials are awaited [10]. The distal section of PT beams, targeting the immediate surrounding layer of tissues at the border of the tumor, might experience a significant increase in LET, causing unwanted toxicities that limit dose escalation and control of radioresistant tumors. Thus, regarding clinical radiobiology, the constant generic 1.1 RBE value, which has been adopted worldwide for PT, might be invalid at the distal part of the Spread-Out Bragg Peak SOBP (≈ 1.4 RBE), in relation to increased LET. 

Despite the limited impact on tumor control, these variations could have a significant impact on toxicity and might contribute to unusual CNS (central nervous system) radiological and clinical abnormalities following PT [11]. Consequently, several reports have stressed the importance of Monte Carlo calculation models, LET cartography, and individual sensitivity with regard to dose escalation and more active treatment of radioresistant cases [12]. Beyond calculation and simulation tools, experimental studies must be performed to accurately compare the bioeffectiveness of protons and other beams and between various types of proton beams, such as scattered beams and the more recent scanned beams. The endpoint could be to express the relationship between beam physics and biology as mathematical models.

Innovations in particle therapy (e.g., robotized gantries and couches, embarked image-guidance, motion-gating, beam intensity modulation, arc therapy, improved calculation algorithms) will allow refined and differentiated tumor coverage, including dose painting (i.e., significant dose escalation) in radioresistant areas, such as hypoxic regions, while sparing normal tissues [13].

However, considering the lack of true enhanced RBE of PT in the tumor, pushing the effectiveness of PT beyond dose escalation will need to consider, as done historically for x-rays, the introduction of combined modalities that specifically radiosensitize tumor cells. Thus, the differential effect between the tumor and extremely close or even tumor-embedded normal tissues and organs (primarily small nervous structures, nerves, and blood vessels) will increase. Moreover, PT could alleviate the global toxicity of combined modalities by dramatically limiting the synergistic systemic toxic effects due to the reduction and near-avoidance of any out-of-field dose [14]. Present experience shows that any chemotherapy that has been validated in combination with x-rays is manageable when coupled with PT [15]. Thus, recent advances are likely to develop further with innovative approaches, such as nanoparticles [16], targeted therapies, and immunotherapy [17,18], as developed in Section 4 of this paper.

To improve our ability to identify eligible patients for advanced PT procedures, especially regarding radioresistant tumors, we must dramatically improve prediction tools, such as the tumor control probability (TCP) and normal tissue complication probability (NTCP) models, advancing toward multiparametric models that incorporate patient- and tumor-specific parameters. To evaluate, consolidate, and validate these models, randomized studies, or at least large cohorts of patients with long-term follow-ups, will be necessary.

### 2.2. Hadrontherapy: Carbon Ions and Multi-Ion Therapy

In addition to prevailing over the physical advantages of protons, carbon ions have greater radiobiological efficacy compared with photons and protons. A strong relationship has been established between ionizing density as measured by the LET and the RBE of ionizing radiation. Low-energy photons (used only for very superficial tumors) and slow charged particles have much higher LET values than the currently used megavoltage photons. The ratio can reach approximately 1000, with values of between 0.3 and 200 keV/µm.

For example, protons that are used for deep-seated tumors have LET values of roughly 0.3 keV/µm (as do megavoltage photons) in their entrance channel and reach approximately 5–20 keV/µm in the distal fall-off region. For carbon ions, these values are ~10 and over 85 keV/µm, respectively. This high LET of carbon ions impacts several biological characteristics of cellular and tissue responses, such as the enhanced killing effect on normal oxygenated tissues (as evidenced by RBE) and hypoxic tissues (based on the OER) that are otherwise extremely radioresistant to low-LET radiation, which has been observed for decades [19].

The ability of high-LET ions to overcome resistance also develops by increasing cell death through the extrinsic ceramide apoptotic pathway [20] and killing telomerase-activated cells [21]. Controversial observations have been reported regarding the level of oxidative stress with high-LET ions in tumor cells. The physiological oxygen tension of the irradiated tissue must be considered to measure the actual oxidative stress that is generated by irradiation at various LETs [22,23]. Hypoxic cells have expressed little or no HIF after ion irradiation compared with photon irradiation, which could be linked to less radioresistance; this observation is also seen under normoxic conditions [23]. These radiobiological responses can enhance the radiosensitivity to ions, in addition to the physicochemical characteristics of their dense ionizing tracks, which can explain the more complex and less reparable DNA damage that is more cytotoxic [24]. A unifying hypothesis of these effects is that more localized, and thus less diffuse, oxidative stress, although dense in the ion tracks, induces complex DNA damage but fewer radiation-induced tumor escape mechanisms.

Tumor cells that are irradiated by ions are less prone to invasion and mobility and switch to a stem cell-like phenotype (CSCs) [25]. CSCs have been implicated in tumor invasion, cancer recurrence, and radioresistance, but how these CSCs should be targeted efficiently remains unknown [26]. Conventional radiotherapy (x-rays) induces an adaptive response in tumors and their microenvironment, which might promote cellular plasticity and thus induce CSC properties in non-CSCs and, ultimately, radiation resistance. This specific response could be mitigated by high-LET irradiation [27].

High-LET beams have enhanced effects on living cells with limited specificity toward tumor cells. Consequently, their application is strictly limited by the tolerance of healthy tissues and is only feasible due to the highly conformal irradiation that is allowed by their finite path and the Bragg peak. However, the unavoidable entrance channel dose is an important parameter of dose and toxicity. The heavier and more highly charged the ion is, the higher the LET will be in the entrance before the Bragg peak. Thus, deep tumors that are treated through long entrance channels must be administered a beam with an entrance LET that is as low as possible while maintaining a high LET in the SOBP. Carbon ions supply a low-LET entrance channel and a high LET in the SOBP. Certain resistant tumors and more superficial tumors could be treated by heavier particles than carbon ions, such as oxygen and neon ions, or combinations of high- and lower-LET particles according to the principles of biologically guided therapy and dose painting. Heavier ions would be preferentially directed to hypoxic tumor areas, whereas lighter ions would deliver the remaining dose to normoxic regions [28]. Overcoming the technical challenges of multi-ion beam sessions is one of the aims for the C400 multi-ion cyclotron (French ARCHADE project).

### 2.3. Ultra-High-Dose-Rate FLASH Proton Therapy

Compared with the conventional RT doses and dose rates in clinical practice (on the order of less 0.5 Gy per second for a 2-Gy session), FLASH radiotherapy (FLASH-RT) uses an ultra-high dose rate (originally described as above 40 Gy/second but more likely reproducible above 100 Gy/second). Initial experiences have been performed with electrons, but photon and proton beams can also be used, provided that they can achieve the instantaneous high dose rate that is needed to observe a FLASH effect. Despite lobbying by proton therapy vendors, it is unknown whether current machine specifications can achieve sufficient instantaneous dose rates. The FLASH effect describes unexpected protection of normal tissue from radiation-induced toxicity in vivo [29]. Due to this sparing effect of healthy tissues, associated with an antitumor effect similar to conventional dose-rate, FLASH-RT is logically considered to be a promising means of increasing the therapeutic ratio [30], including with proton therapy. The underlying mechanisms remain incompletely understood, although a notable hypothesis is being tested and despite the clinical requirements for treating small-field superficial tumors and deep tumors using multiple beams having not been tested [31].

## 3. Role of Microenvironment and Surrounding Healthy Tissues in Tumor Radioresistance

### 3.1. Hadrontherapy to Overcome Hypoxia-Induced Radioresistance?

One of the prevailing mechanisms of radioresistant tumors is hypoxia. Clinical investigations that have been performed since the 1950s have demonstrated that the presence of hypoxic tissue (areas with O_2_ tension (pO_2_ values) ≤ 2.5 mmHg) is one of the hallmarks of tumors and an important factor of its pathophysiology and treatment resistance. Up to 60% of locally advanced solid tumors primary and metastatic tumors [32] bear hypoxic or anoxic tissue that is distributed heterogeneously throughout the tumor mass. Hypoxic or anoxic areas arise due to an imbalance between the supply and consumption of oxygen.

There are several major mechanisms in the development of hypoxia in tumors: (i) severe structural and functional abnormalities of tumor microvessels (perfusion-limited O_2_ delivery), (ii) limited diffusion of O_2_ delivery, and (iii) tumor-associated and therapy-induced anemia, leading to reduced availability of O_2_ (from blood). These mechanisms lead to substantial heterogeneity in tissue oxygenation in the tumor.

Protons have a patent ballistic advantage over photons: their RBE is equivalent to that of photons, and oxygen depletion influences the tumor response to proton therapy [33]. At low and high doses of radiation, cell death increases under oxic versus conditions, engendering the concept of the oxygen enhancement ratio (OER).

OER is quantified as the ratio of doses that are required to achieve the same biological effect under hypoxic or normoxic conditions. The oxygen effect, quantified by OER, decreases with rising LET, suggesting a clinical advantage of high-LET radiotherapy with heavy ion beams, such as carbon therapy, compared with low-LET photon and proton irradiation [34,35]. For anoxic cells that are irradiated with photons, the OER is approximately 3 for a surviving fraction (SF) of 10% [36]. For carbon ions with low LET in the entrance channel, the OER is roughly the same as for photons, whereas with increasing LET, the OER declines and reaches 1 at high dose-averaged LET values of ≈500 keV/µm [37,38]. The sensitivity of cells to intermediate- and high-LET carbon ion irradiation is less dependent on oxygen tension compared with photon irradiation. Carbon ion RT is thus assumed to result in better control of hypoxic tumors. However, the LET of the therapeutic dose in tumors is usually 30–80 keV/µm, which yields an OER of 2–3 [37], at which an oxygen effect remains [39,40].

Notably, carbon ion RT can also overcome hypoxia [39] through the induction of complex DNA damage by densely packed ionization [41]. Following exposure to ionizing radiation, if molecular oxygen is present, DNA can be damaged directly by radiation or indirectly through ROS from the radiolysis of water, enzyme-mediated ROS production, and altered aerobic metabolism. Under hypoxic conditions, DNA damage that is induced by low-LET radiation can be more readily repaired. DNA radical levels can be reduced by sulfhydryl groups (SH groups), rendering single- and double-strand DNA breaks less severe under hypoxic conditions. Direct action due to high-LET radiation is less affected by the presence of oxygen [34]. Other hypotheses include the formation of interacting radicals, increased lesion complexity [42], and radical multiplicity [43].

A dose-dependence might exist between OER and dose per fraction [44]. The ‘‘oxygen-in-the-track’’ hypothesis could explain the inverse relationship between OER and LET. Using Monte Carlo track simulations of pure and de-aerated water, it has been demonstrated that carbon ion radiation increases oxygen concentrations substantially in the tracks of these high-LET particles, quickly leading to water radiolysis (between 10^−12^ and 10^−5^ s). Thus, the amounts of oxygen that is locally produced are sufficient to decrease OER with rising LET [45]. This effect is attributable to the sole radiolytic formation of O2 in the tracks of carbon ions, and an ‘‘oxygenated’’ microenvironment around the particle track has also been observed in cells that have been irradiated under anoxic conditions [46].

In addition to chemical mechanisms, there is evidence that implicates biological factors, such as cellular enzymatic repair processes, in explaining why the OER declines as LET increases [45]. The expression of several transcription factors, including the heterodimeric hypoxia-inducible factor-1 (HIF-1), is regulated by hypoxia. HIF-1 correlates with a poor prognosis, local recurrence, distant metastases, resistance to chemotherapy and radiotherapy [47,48] through its hypoxia-induced α-subunit. Carbon ion RT downregulates HIF-1 signaling in lung and head and neck models [49]. HIF activation depends on ROS production, primarily HO• hydroxyl radicals, and is independent of an oxygen effect [23,50]. The dense and homogeneous distribution of ROS (HO•) following x-rays activates migration/invasion mechanisms, whereas the clustering of ROS around carbon ion trajectories can prevent HIF-1 and upstream signaling [50]. Targeting HIF-1 or its target genes, such as survivin and erythropoietin receptor, could enhance the efficacy of photon RT and carbon RT in several tumor types [23,49,51,52].

In summary, hadrontherapy using high-LET irradiation can overcome the intrinsic radioresistance of hypoxic tumors. Carbon ion RT might be more efficient than proton therapy and other types of low-LET radiation for hypoxic tumors through a reduction in the oxygen effect, based on the relationship between pO2, LET, and OER, DNA repair complexity, and biological pathway activation [53,54]. Combinations of hadrontherapy with oxygen-dependent and DNA repair pathway-interacting molecules should be examined further in preclinical and clinical studies. Notably, hypoxia also influences the interaction between cancer cells and immune cells by downregulating MHC class I molecules, and several immune checkpoints are upregulated by hypoxia.

### 3.2. Hadrontherapy to Reverse Tumor Microenvironment-Induced Immune Tolerance

Radioresistance is a matter of not only intrinsic tumor cell radiosensitivity and hypoxia but also the immune microenvironment. Recruitment of infiltrative immune cells, such as tumor-infiltrating lymphocytes (TILs) and tumor-associated macrophages (TAMs), is frequently observed in tumors. They have tumor-specific prognostic and predictive values in many malignancies [55], and immune escape is one of the hallmarks of cancer [56]. There is a complex tumor-dependent balance between immune cell infiltration and activation or repression of the immune system to promote tumor growth. Immune cells can reside in the core of the tumor, at the invasive front, and in adjacent tissues (see for review [57]). Among infiltrating cells, the major cells that are involved include macrophages (referred as TAMs), which are central to controlling antitumor immunity [58]. TAMs are more often associated with the M2 phenotype [59], which usually correlates with a poor prognosis, whereas the M1 phenotype is linked to a better prognosis in tumors [60,61,62].

Lymphocytes, natural killer cells, and dendritic cells are also significant in tumor inflammation. Chronic inflammation influences malignant transformation, and proinflammatory molecules are associated with the induction of hyperproliferative signaling, resistance to cell death, induction of evasion, and metastasis.

Although the function of inflammation in tumor growth is now well established, recent studies have focused on radiation-induced inflammation that can mediate radioresistance. External factors, such as conventional radiotherapy, regulate the fate of macrophages in tumors [63,64]. RT can alter the tumor microenvironment by inducing a favorable niche for M2 macrophages in the junctions between central necrotic and surrounding hypoxic regions of tumors [65]. Enrichment in M2 macrophages in hypoxic tumors in cell lines [64] and rodents [66] after irradiation has been interpreted as the result of the preferential death of M0/M1 macrophages [66]. Such selective depletion might be due to greater radiosensitivity of M0/M1 versus M2 macrophages [66]. Furthermore, Teresa-Pinto et al. demonstrated that irradiation induces proinflammatory and anti-inflammatory markers on human macrophages from peripheral blood [67].

Exposure to radiation activates several transcription factors that control the expression of various molecules that supporting cancer progression. For example, stimulation of NF-kB in inflammatory cells results in the activation of various cytokines, such as IL10 and TGFb, which in turn enhance proliferation, invasion, and vascularization.

Considering that irradiation induces immunosuppressive mechanisms, the results above prompted the scientific community to examine the effects of other RT modalities in stimulating antitumor inflammation and thus reducing radiation-induced inflammation

In healthy rats, whole-body carbon therapy significantly increased NK activity from 0 to 0.05 Gy and decreased it from 0.05 Gy to 2 Gy. This study suggested a stimulatory effect of carbon ion radiation on immune cells [68]. In the context of tumors, conditioned medium from irradiated U87-MG glioblastoma cells stimulated THP-1 and Jurkat cell proliferation and THP-1 differentiation in macrophages [69]. Other effects included upregulation of PD-L1 in osteosarcoma cells after carbon ion irradiation, as observed with x-rays [70]. Conversely, in prostate cancer, a carbon ion-treated patient experienced a decrease in tumor cells and rise in CD4+ cells and CD4+/CD8+ ratio during the RT and until 1 month after treatment. Secreted cytokines have been proposed to interact with immune cells, increase CD4+ cell proliferation, and enhance antitumor immunity [71]. Similarly, the levels of proinflammatory cytokines rise after carbon therapy compared with x-ray irradiation in RAW264.7 macrophages [72]. Reduced macrophage M2 polarization and increased CD8+ inflammation have been observed in vivo in a syngeneic orthotopic glioblastoma mouse model after carbon irradiation [73].

In summary, preclinical investigations suggest that carbon ion irradiation induces a proinflammatory response that increases its antitumor efficacy compared with photons and protons. However, little is known about inflammation and radioresistance in the context of hadrontherapy, and further studies on various tumor locations, immune cell types, and radiation modalities are awaited.

### 3.3. Contribution of Bystander Effects to Normal Tissue Tolerance in Hadrontherapy

The intercellular communication that is triggered by damaged irradiated cells might unbalance the physical accuracy of accelerated ions by a biological imprecision, contributing to the side effects of radiation. Irradiation can induce a biological response in nonirradiated cells that are near irradiated cells [74]. This effect, termed radiation-induced bystander effect (RIBE), depends primarily on the cell type, irradiation quality, and dose [75]. RIBEs are defined as genotoxic effects in nonirradiated cells [76], in close proximity (bystander) or in other regions of the same organ or even different organs (abscopal) [75,76,77]. Genotoxic effects include DNA damage, chromosomal aberrations, mutations, and apoptosis [74], arising due to complex damage-sensing mechanisms that result in DNA repair, delays in the cell cycle, or cell death [78]. Nuclear DNA is not the sole target of RIBEs—the cytoplasm can be affected, causing genotoxic effects (e.g., micronuclei) in neighboring cells [79]. Metabolic ROS [80] and irradiation-induced ROS and nitric oxide (NO) [81,82] are important in the redox-sensitive signaling pathway in bystander cells. Ca^2+^ influx can also induce RIBEs by increasing the permeability of the mitochondrial membrane and subsequently inducing apoptotic cell death after high-LET or low-LET radiation.

Bystander effects depend on various physical factors, such as the total absorbed dose, dose rate, biological characteristics of the target (dimensions, sensitivity, regeneration, etc.), and distribution of radiation sources and structure. Using proton mini-beams, early apoptotic events have been induced in normal keratinocytes that have been exposed to medium from irradiated cells or are in the vicinity of irradiated cells, independent of dose or the number of protons that were delivered. Three-dimensional (3D)-based mathematical models and clinical data could help us understand the mechanisms of RIBEs [83,84,85]. Differences in RIBEs have been observed between x-ray, gamma-ray irradiation, and hadrontherapy with carbon ions. Although the RBE is 2–3 times higher for carbon ions versus x-rays, the RIBEs are similar in magnitude [86,87].

A bystander effect was observed when transferring the conditioned medium of irradiated chondrosarcoma cells to chondrocytes, but the reduction in proliferation was lower with C-ions compared with x-rays at the same doses [86]. In contrast, in bystander cells, the induction of micronuclei and cell death increased with LET when conditioned medium from irradiated cells was transferred to bystander cells deficiencies in DNA repair [88]. In addition, chronic oxidative stress was observed in bystander cells that were cocultured with irradiated cells, even after 20 generations, as a function of LET [89].

Although RIBEs have been demonstrated in vitro, the gap in response remains too large to estimate their magnitude in vivo during radiotherapy. It is challenging to visualize and quantify bystander/abscopal effects in animals and estimate their contribution to damage to nonirradiated normal tissues. Mathematical models suggest that a substantial proportion of the response to radiation therapy results from intercellular communication rather than direct damage, with significant differences between the signaling-adjusted and physical doses [90]. According to such models, organs in low-dose regions near the target volume experience the largest increases, with mean signaling-adjusted doses rising from 23 to 33 Gy [90].

RIBEs increase intercellular communication between irradiated and nonirradiated cells and appear to be LET-dependent [89,91]. Because RIBEs might be lower with high-LET irradiation [86], further data on RIBEs with hadrontherapy (high- and low-LET) versus x-rays are needed.

### 3.4. Tumor Control and Normal Tissue Tolerance in Hadrontherapy

The physical deposition of doses of protons and heavier ions renders them attractive, based on their ability to spare healthy tissues and, for the latter, increase the antitumor efficacy. However, whereas the dose distribution spares healthy tissues better in the entry channel better compared with megavoltage photons, a significant proportion of the dose is deposited in normal tissues in front of the tumor. Moreover, the fragmentation tail of heavy ions comprises secondary particles, which differ in terms of properties of LET and biological effects and which deliver a small amount of the dose beyond the target.

Recent reports have shown a decrease in early and late toxicity with proton therapy compared with conventional irradiation with photons: Romesser et al. [92] observed that proton therapy for head and neck cancers lowered the rates of early moderate toxicities by 5-fold (10% instead of 50%); Yock et al. [93] reported ototoxicity and neuroendocrine deficits but no other toxicities after proton therapy of medulloblastomas, with a median follow-up of 7 years. With respect to cancer induction, Chung et al. [94] recorded 5.2% of proton therapy patients having secondary tumors compared with 7.5% for photons in a series of over 1000 patients. The risk of secondary malignancies due to proton therapy was also lower in Sethi et al. [95].

With carbon ion therapy (for review, see [96]), the toxicities in bone and soft tissue sarcomas decreased compared with conventional radiotherapy [97]. However, skin reactions can be substantial [98,99,100,101]. Other severe toxicities have been reported in series of advanced tumors in approximately 20% of patients [102,103], but clinical trials remain ongoing [96]. Concerning secondary malignancies, Mohamad et al. suggested that secondary cancers can be reduced with carbon RT [104]. There is a strong need for comparative studies between hadrontherapy and conventional radiotherapy to determine the efficiency of hadrons in killing tumor cells and evaluate the toxicity in normal tissues. In particular, nontargeted effects might mitigate the sparing effects of hadrontherapy on normal tissues.

## 4. Biomarkers for Guidance of High-Precision Radiation Therapy

### 4.1. Liquid Biopsies and Precision Ion Therapy to Monitor Response of Tumor and Healthy Tissue

With regard to the tumor response, conventional imaging might be insufficiently sensitive to observe the changes that are induced by various radiation modalities; circulating biomarkers of tumor growth, oxidative stress levels, and inflammatory responses can provide additional data and constitute early surrogates of the tumor response to hadrontherapy compared with conventional photon-based irradiation. A biomarker is defined as ‘‘any substance, structure, or process that can be measured in the body or its products and influence or predict the incidence or outcome of diseases’’ [105,106,107,108]. In radiation research, this classification includes biomarkers of exposure, susceptibility, late effects, and persistent effects [109].

At the cellular level, the response to radiation modalities appears to differ due to the quality and quantity of the resulting DNA damage, for example—not to the particle itself. Among protein biomarkers, such as pP53, pATM, MDM2, and XPC, only p-DNA PKC is upregulated after alpha versus gamma radiation [110]. Perhaps by examining the complexity of DNA damage using, for example, gamma-H2AX foci in combination p-DNA PKcs, one could estimate the quality of the radiation that targets the cell. The effects of radiation can also be compared and followed in vivo in cancer patients by monitoring serum thymidine kinase, which is indicative of proliferative ability.

In treated patients, once an organ is irradiated, various types of molecules, such as miRNA, proteins, and damaged dNTP, are released from the irradiated tissue into the bloodstream or other body fluids. Circulating biomarkers could be used to detect, analyze, and monitor treatment effects in blood, saliva, or urine instead through a biopsy of the target tissue. Examples of such molecular biomarkers are 8-oxo-dG (a stress biomarker), proinflammatory-related biomarkers, and thymidine kinase 1 (TK1). These molecules can be detected directly in the bloodstream as a measure of oxidative stress, immune response, and tumor growth, respectively. Some of these factors can be detected in other body fluids. This section focuses on the potential for circulating biomarkers to determine proliferation rates, proinflammatory responses, and oxidative stress levels. These biological pathways have been related to poor outcomes and resistance to radiotherapy [111,112,113]. However, most knowledge on circulating biomarkers in radiotherapy is based on classical photon radiotherapy, and few have been validated in vivo in response to hadrontherapy.

#### 4.1.1. Tumor Growth

Most tumor-related biomarkers are not diagnostic but are useful for monitoring the outcome after oncological treatments.

Biomarkers that indicate tumor proliferation rate, such as thymidine kinase 1 (TK1), can be determined in tumor tissue (tTK1, immunohistochemistry) and in body fluids, such as serum (STK1). TK1 in serum can be quantified by its activity (TKa) or concentration (STK1p). STK1p is valuable for nearly all tumor types, whereas TKa is useful primarily for lymphoma, leukemia, and certain solid tumors (breast, lung). Because the concentration of TK1 in serum is low (≈0.5–20 pM), a sensitive enhanced chemiluminescent immune dot blot assay (ECL dot blot) was developed.

tTK1 and STK1p correlate to the prognosis (survival, relapse) and treatment effects. Over 10,000 cancer patients with 20 tumor types have been tested in clinical trials, and more than 50,000 people have undergone health screens [114,115,116]. By ECL dot blot, TK1 can indicate the risk of relapse earlier than imaging techniques and thus might be useful for monitoring treatment effects. There are no data on the use of TK1 in hadrontherapy. However, it would be interesting to determine the effects of hadrontherapy, based on the change in TK1 levels.

#### 4.1.2. Inflammatory Response Biomarkers

During massive necrotic death of cancer cells and surrounding tissue cells, radiation triggers inflammatory reactions that are analogous to the wound-healing response [111] and stimulate cell proliferation and survival, which can lead to radioresistance and relapse. Furthermore, the eicosanoid pathway, associated with cyclooxygenases (COXs), and lipoxygenases (LOXs) can be activated by radiation and contribute to inflammation and cell proliferation and survival after irradiation [117]. However, in certain cases, therapy-induced inflammation, when induced particularly by particle irradiation, can enhance antigen presentation, leading to immune-mediated tumor cell death [118,119]. This mechanism bridges innate and adaptive immunity, initiating local and systemic antitumor responses [120]. Notably, particle irradiation increases the multifunctional ceramide pathway that is involved in NF-κB activation more efficiently than photon irradiation [121].

Irradiation of tissue by photon, proton, and carbon ions leads to the release of free radicals and the activation of NF-κB at various levels, a transcription factor in several immune signals, such as TNF-alpha, IL1, and IL6 [122,123,124]. Furthermore, the release of signaling molecules affects the recruitment of proinflammatory cells to the site of damage and their activation. Such cells produce free radicals, which react with various biomolecules, altering their structures and disrupting their function.

The expression of radiation-induced cytokines might occur immediately after exposure and continues as a cascade that persists for months and possibly years after the exposure is completed. Proinflammatory interleukin 1beta (IL-1β) and TNF-alpha and innate immune proteins, such as pentraxin 3, are upregulated in the arteries of patients who have been treated with photon radiotherapy [125]. Inflammatory processes in the body after exposure to any type of ionizing radiation can be studied using set of plasma biomarkers, including CRP, PTX3, TNF-alpha, NF-kappa B, IL-1b, and IL10.

#### 4.1.3. Oxidative Stress Biomarkers

Exposure to ionizing radiation and other toxic endogenous and exogenous agents can activate the stress response. Efficient activation of an oxidative stress response might be required for optimal response to radiotherapy. Pre-existing high oxidative stress levels can influence this response [126,127]. Most indirect DNA damage that is induced by radiation arises from hydroxyl radicals that are produced from the interaction of ionizing radiation with water [128,129]. However, ROS can also be released from dysfunctional mitochondria that are induced directly by high- and low-LET irradiation [130,131,132].

Among the many types of nucleic acid modifications that are elicited by ROS during oxidative stress, 8-oxo-dG has been used widely as a sensitive marker of general systemic oxidative stress in vivo and in vitro [133,134,135]. A recent study found that that esophageal and gastric cancer patients with lower oxidative stress before the start of radiotherapy, as measured by 8-oxo-dG in the serum, had a better prognosis than those with higher levels. Similar results on the prognostic value of extracellular 8-oxo-dG have been seen in lung cancer [136,137], colon cancer [138], and oral cancer [139]. Extracellular 8-oxo-dG might be a biomarker that predicts the sensitivity of cancer patients to radiotherapy. However, these in vivo studies were performed when patients received photon radiotherapy; there are no data on 8-oxo-dG in patients who underwent hadrontherapy.

### 4.2. Functional/Metabolic Imaging of Tumor Tissue and Its Microenvironment

A significant factor in the origin of radioresistance is tumor heterogeneity with a tumor subvolume that is at high risk of relapse—particularly for a region with pronounced hypoxia. Physiologically, human tissues have a tissue pO_2_ (ptO_2_) of 30 mm Hg to 70 mm Hg, referred to as normoxic. Hypoxia, generally defined when ptO_2_ falls below 10 mm Hg, is the result of unbalance between oxygen consumption and delivery.

However, tumor hypoxia is highly heterogeneous over time and by location. One of the main explanations proposes two overarching types of hypoxia: diffusion-limited hypoxia, also referred to as chronic hypoxia, and perfusion-limited hypoxia, or cycling hypoxia [140]. Because hypoxia reduces the efficacy of RT, various efforts have been made to map hypoxia and then tune the dose deposition as a function of oxygen concentration. This concept was introduced in the early 2000s [141] and was then adapted to hypoxia by introducing the concept of “hypoxia dose painting” [142].

Consequently, a good hypoxia imaging tool in the context of tumor and radiation therapy should fulfill various requirements. It should distinguish normoxia from hypoxia and anoxia and differentiate between perfusion-limited and diffusion-limited hypoxia. Thus, such tools should have excellent temporal and spatial resolution, with regard to which biomedical imaging tools have demonstrated great advantages [143]. This is particularly true in radiation therapy, when the treatment is suspected of inducing changes in oxygenation in the tumor and for which a repeat examination is needed. Among the various imaging biomarkers that have been designed [144], PET and Magnetic Resonance Imaging (MRI) comprise surrogate biomarkers of hypoxia. PET markers have good sensitivity and specificity for hypoxia, whereas MRI can provide excellent spatial resolution, which could be advantageous for boosting hadron-based radiotherapy.

Increasing radiotherapy to the most hypoxic regions has not been addressed and deserves further study.

To target a region that is at high risk of recurrence, the detection of areas with a high proliferation rate is important, due to the elevated RBE of carbon ions. In a retrospective study, MET-PET volumes were suggested to be predictive of survival, regional control, and distant control following carbon ion radiotherapy [145]. An active clinical trial is also ongoing to boost the biological effect in the most proliferation region of the tumors, based on a combination of advanced MRI and PET imaging biomarkers [146].

## 5. Combined Strategies to Improve Particle Therapy

### 5.1. PARP Inhibitors in Association with Radiotherapy

Because particle therapy reduces the volumes of irradiated normal tissue compared with photons, any combination of proton or carbon therapy could help fully exploit the radiosensitizing effects of most systemic drugs and increase the differential effect.

PARP are proteins that recruit factors in important cellular processes, such as modulation of chromatin structure, transcription, replication, recombination, and DNA repair. PARP-1, -2, and, to a lesser extent, PARP-3 have been implicated in most steps of the DNA damage response [147,148]. They recognize DNA damage, recruit single-strand break (SSB) and base excision repair (BER) factors, facilitate chromatin relaxation and the access of DNA repair agents, and favor accurate homologous recombination when nonhomologous end-joining (NHEJ) is intrinsically mutagenic.

Due to synthetic lethality, PARP inhibitors are highly effective as a single antineoplastic agent in patients whose tumors have germline or somatic defects in DNA damage and repair genes (e.g., ATM, BRCA1, BRCA2) or defects in genes that are involved in phosphatase and tensin homolog (PTEN) signaling. Targeting cells with a specific DNA repair defect by inhibiting a second DNA repair pathway with PARPi is the one of the best example of “synthetic lethality” [149,150].

Inhibition of PARP activity also leads to tumor cell radiosensitization in vitro and in vivo. Defects in specific DNA repair pathways enhance the radiosensitizing effects of PARP inhibition. In addition to inherent genetics, tumor cells can be preferentially sensitized to radiotherapy through various mechanisms, including proliferation-dependent radiosensitization, targeting of the endothelium and tumor vasculature, and increased sensitivity to PARP inhibitors in repair-deficient hypoxic cells [151].

The radiosensitizing effect of PARP inhibitors (PARPi) increases the ratio of irradiation effectiveness to 1.05 and 2.87, depending on the cell line, oxygen conditions, and irradiation modality [152].

Particle therapy might be the best irradiation modality to combine with PARPi. Due to the moderate integral dose that is delivered to self-renewing surrounding normal tissues (e.g., skin, digestive tract), with particle irradiation modalities [153,154,155], the toxicity of the combination is expected to be lower. Furthermore, the radiosensitizing effects are higher in extent in the Bragg peak than in the entrance region [156], which allows the therapeutic ratio of PARPi as a radiosensitizer to be increased. Finally, high-LET irradiation induces more complex DNA damage than photons—particularly, oxidative clustered DNA lesions that comprise oxidized bases, apurinic-apyrimidinic sites, and SSBs. This damage is essentially repaired by BER, for which PARP is critical. Consequently, the inhibition of PARP delays the repair of oxidative clustered DNA lesions and favors their conversion to highly lethal DSBs [153,154,157].

The combination of PARPi and hadrontherapy has shown promise in preclinical studies, and human phase I studies of the combination of PARPi and particle therapy are awaited.

### 5.2. Nanoparticle-Enhanced Radiation

The combination of radiotherapy can systemic agents can increase the radiation efficacy—i.e., it can radiosensitize tissues, and therefore, be used to overcome radioresistance. The drawback of such a strategy is the systemic toxicity of radiosensitizing agents, such as chemotherapy and targeted therapy. Radioenhancing nanoparticles are usually formed from heavy chemical elements in the metal category of Mendeleyev’s table. They are biocompatible but are inert and nontoxic without irradiation. Nanoparticles have thus appeared as a method of enhancing the radiation efficacy in radioresistant tumors while preventing systemic side effects. Nanoparticles improve the efficacy of radiation through photoelectric and Compton effects, producing a dense core of secondary electrons (photoelectrons and Auger electrons) around the tumor cells on the micrometric scale [158]. The resulting dose enhancement factor is between 2 and 9, depending on the study and dose calculation algorithm.

The diffusion and retention of these nanoparticles in the tumor often rely on a passive phenomenon (enhanced permeability and retention, EPR) that is related to the tumor microenvironment and their characteristics. To increase the intratumoral concentration of nanoparticles (and thus the dose enhancement ratio), nanoparticles can be optimized by functional coating (with motifs that target the tumor components) or injected into the tumor.

Whereas most clinical irradiation is performed in the megavoltage range with photons to treat deep tumors, new modalities of irradiation could be more efficient with nanoparticles in radioresistant tumors. Preliminary experiments have shown that extremely destructive phenomena, such as the Mössbauer effect, can be achieved at the energy levels of the photoelectric effects (several tens of keV) using synchrotron radiation [159].

Because proton therapy has an average biological effectiveness that is similar to conventional irradiation, nanoparticles might also be useful in combination with proton therapy for radioresistant tumors [16]. Clinical trials on the combination of proton therapy and nanoparticles are being prepared. Some works also take original strategies, such as using protons without a spread-out Bragg peak (SOBP), because the dose-increasing factor can be high [160]. Possible interactions between particle therapy and nanoparticles differ from those of photons and could be triggered by projectile-induced x-ray emission (PIXE), which initiates intra-atomic cascades of electron emissions. Imaging and range uncertainties are being assessed using Monte Carlo algorithms.

Gold nanoparticles are the most commonly studied nanoparticles in vitro but are seldom used in clinical studies due to their redox properties, which confer poor compatibility. Various nanoparticles have entered clinical application. NBTRX3 oxide hafnium nanoparticles by Nanobiotix have demonstrated clinical efficacy in sarcomas in a phase 3 trial [161]. They increased the complete pathological response rate by 2-fold and the complete resection margin (R0) rate. Thus, the first treatment authorization has been obtained for radioenhancing nanoparticles for this indication. These nanoparticles are also used in liver and head and neck tumors and oligometastases. Other nanoparticles have been used with a theranostic approach (i.e., with imaging and therapeutic aims), such as gadolinium nanoparticles [162]. Several early-phase trials have been initiated (see ClinicalTrials.gov).

### 5.3. Association of Immunotherapy with Proton and Carbon Radiotherapy

Immunotherapy has emerged as an effective systemic treatment for many solid cancers, with objective responses in up to 25% of patients [163]. Ionizing radiation can synergize with immunotherapy [164]. Proton and photon irradiation can induce immunological cell death (ICD) and immunogenic modulation in the tumor. Compared with photon radiation, proton radiation enhances killing by T cells by upregulating their cell surface expression of calreticulin [165]. In addition, the RBE of high-LET particle radiation can induce robust ICD and immunogenic modulation [18].

Sparing nonmetastatic lymph nodes and reducing irradiated volumes is a potentially new approach to reduce radiation-induced lymphopenia and improve the antitumor immune response [166]. Radiation in the lymph nodes can abrogate the priming of T cells by dendritic cells, and thus, impair the antitumor immune response. Immunotherapy can treat potential nonirradiated microscopic tumor cells. Consistent with this strategy, the physical advantage of low-LET proton radiation and the physical and biological benefits of high-LET particle radiation could be exploited to spare mediators of the immune system, such as T cells, and favor synergy with immunotherapies [167].

## 6. Conclusions

Research on tumor radioresistance encompasses many scientific domains, from radiation physics to radiobiology and radiation therapy. Individualized medicine can now apply optimized delivery of radiation, such as ultra-high-dose-rate irradiation and multi-ion therapy, to provide tumor dose painting and normal tissue sparing due to better physical and biological properties. Such properties also allow the development of innovative drug-radiotherapy combinations and can be monitored with noninvasive biomarkers.
